# Bronchopleural Fistula Resolution with Endobronchial Valve Placement and Liberation from Mechanical Ventilation in Acute Respiratory Distress Syndrome: A Case Series

**DOI:** 10.1155/2017/3092457

**Published:** 2017-03-07

**Authors:** Haris Kalatoudis, Meena Nikhil, Fuad Zeid, Yousef Shweihat

**Affiliations:** ^1^Pulmonary Department, Byrd Clinical Center, Marshall University School of Medicine, 1249 15th Street, Huntington, WV 25701, USA; ^2^Internal Medicine Department, Marshall University School of Medicine, 1600 Medical Center Drive, Huntington, WV 25701, USA

## Abstract

Patients who have acute respiratory distress syndrome (ARDS) with persistent air leaks have worse outcomes. Endobronchial valves (EBV) are frequently deployed after pulmonary resection in noncritically ill patients to reduce and eliminate bronchopleural fistulas (BPFs) with persistent air leak (PAL). Information regarding EBV placement in mechanically ventilated patients with ARDS and high volume persistent air leaks is rare and limited to case reports. We describe three cases where EBV placement facilitated endotracheal extubation in patients with severe respiratory failure on prolonged mechanical ventilation with BPFs. In each case, EBV placement led to immediate resolution of PAL. We believe endobronchial valve placement is a safe method treating persistent air leak with severe respiratory failure and may reduce days on mechanical ventilation.

## 1. Introduction

Persistent air leak (PAL) due to alveolopleural or bronchopleural fistulas is associated with an increased length of stay, cost of care, and morbidity [1, 2]. Critically ill patients who develop bronchopleural fistulas (BPF) tend to have prolonged hospital admissions with a poor prognosis [3]. Air leak from BPFs reduces effective minute ventilation and oxygenation [4]. In acute respiratory distress syndrome (ARDS), BPFs can cause incomplete lung expansion, loss of effective tidal volume and positive end expiratory pressure (PEEP), and reduced carbon dioxide elimination [5]. Using endobronchial valves (EBV) should be considered a safe and effective option to treat BPFs [6].

High airway pressure is commonly used in ARDS to ensure continued patency of the fistula; however, this may impair healing and thus closure of the fistula. Endobronchial valves (EBV) are small unidirectional devices that allow air to escape when placed in the segmental or subsegmental airway [7]. This will prevent air from entering the fistula and result in atelectasis and collapse of the fistula. The process of recovery would lead to fibrosis with resolution of the shunt and eventual extraction of the valve. Consequently, this will increase the effective tidal volume and retained pressure delivered by the mechanical ventilator, hence improving oxygenation and ventilation.

We present three cases that required prolonged mechanical ventilation with persistent bronchopleural fistulas with acute respiratory distress syndrome that were successfully extubated soon after endobronchial valve placement.

We describe three cases where EBV placement facilitated endotracheal extubation in patients with severe respiratory failure on prolonged mechanical ventilation with BPFs.

## 2. Patient 1

**Figure 1:**
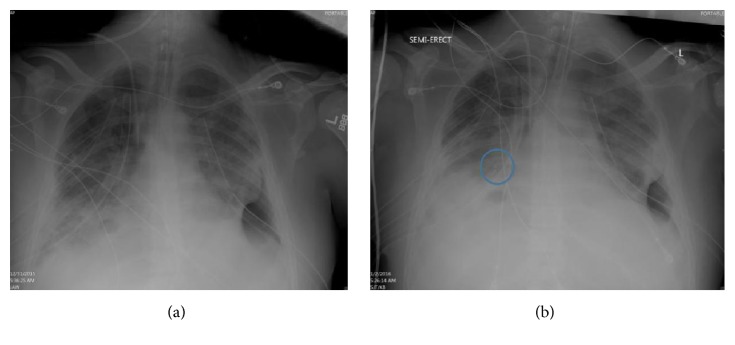
The left chest roenterogram represent pre-EBV placement. The right chest roenterogram represents post-EBV placement. The circled area depicts the EBV with right middle lobe atelectasis. EBV: endobronchial valve.

A 32-year-old male presented with worsening dyspnea, fevers, and cough for two weeks. In the ED he developed respiratory failure and was immediately intubated. A chest roentgenogram showed complete left-sided opacification with mediastinal shift to the right. Emergent chest tube diagnosed and relieved a large loculated empyema. It was further treated with fibrinolytics (tissue plasminogen activator) and dornase alpha since surgery was nonoptional due to multiorgan failure and septic shock. His condition continued to worsen when he developed a right-sided pneumothorax requiring a total of three chest tubes. A large volume air leak persisted despite low tidal volume ventilation with attempts to reduce the peak pressures as well as the plateau pressures. Conservative management failed and he was again deemed not to be a surgical candidate to correct the air leak. On mechanical ventilation day 15, he was evaluated for an endobronchial valve. His respiratory acidosis continued to increase and his PF ratio (PaO2/FiO2) was 150 and decreasing with worsening bilateral infiltrates. Balloon occlusion of the right middle lobe eliminated the air leak. Subsequently, EBV (Spiration Valve Systems, Olympus, USA) implantation completely sealed the BPF. This caused right middle lobe atelectasis (Figure 1). He was liberated successfully from the vent 5 days after the cessation of the leak. He was evaluated 6 weeks later and the EBV was removed without any complications.

## 3. Patient 2

**Figure 2:**
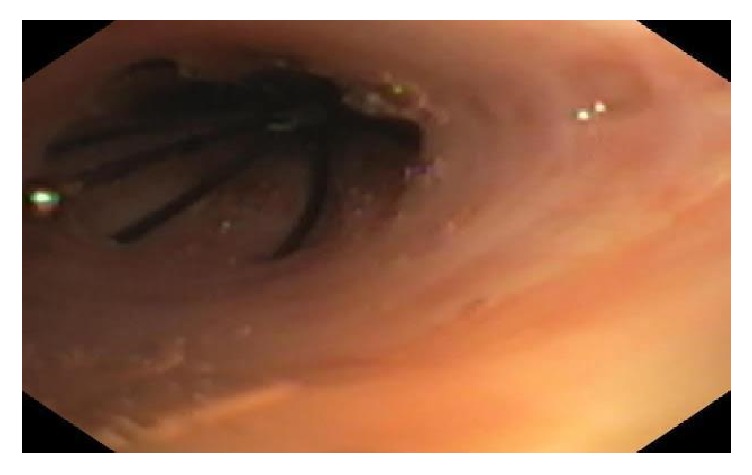
One of two endobronchial valves paced within the right middle lobe.

A 43-year-old female presented with productive cough, hematochezia, fatigue, and weight loss of 15 pounds was found to have a left lower lobe abscess (5 × 2.8 cm) and right middle lobe abscess (4 × 3 cm). She was admitted into the ICU and treated for septic shock due to bilateral pulmonary abscess appropriately. Bilateral spontaneous pneumothorax occurred and she experienced an obstructive cardiac arrest requiring bilateral needle decompression to achieve return of spontaneous circulation. The patient developed bilateral fungal empyema that required chest tube drainage at multiple sites; as a result, a persistent air leak developed on the right side. The patient developed transfusion associated lung injury after she received packed red blood cells. Her lung injury improved but she failed multiple weaning trials that were believed to be secondary to the BPF. Due to multiorgan failure, surgical intervention was deferred to close the BPF. On mechanical ventilation day 11 and following a successful balloon occlusion test, two EBVs (Spiration Valve System, Olympus, USA) were placed in the right middle lobe with complete resolution of BPF (Figure 2). The patient was successfully extubated three days later. Patient survived to hospital discharge but was lost to follow-up there after.

## 4. Patient 3

A 38-year-old female smoker arrived with bilateral infiltrates and severe hypoxic respiratory failure needing mechanical ventilation. An iatrogenic pneumothorax developed that required chest tube placement. She developed an air leak with worsening oxygenation. Bronchoscopy was done with transbronchial biopsies. The biopsy revealed organizing pneumonia. After she was given steroids, the gas exchange improved rapidly; however, she failed weaning trials due to the persistent air leak. On mechanical ventilation day 9, balloon occlusion showed resolution of the leak. Seven EBVs (Spiration Valve System, Olympus, USA) were placed in the right upper and middle lobes. Multiple valves were required in two lobes to occlude all segments to completely abolish the leak. This was most likely due to multiple defects in multiple lobes. She was extubated on day 13. The valves were removed at 4 weeks after the placement without any complications.

She continues to do well after discharge.

## 5. Discussion

Diagnosis and management of a BPF should occur in a stepwise fashion [8]. Initial treatment with chest tube drainage of pneumothorax with persistent air leak usually continues for a greater period of time when there is underlying pulmonary disease [9]. In the spontaneously breathing patient, surgical intervention can be attempted if the BPF is prolonged. Other options may include Heimlich valve or pleurodesis if the lung remains inflated on chest X-ray [10]. The American College of Chest Physician expert panel recommends that a BPF should be observed for five days prior to intervention, such as EBV placement, in nonsurgical candidates [11]. In patients who require mechanical ventilation, conservative management of BPFs, such as a reduction (or elimination) of PEEP, effective tidal volume, and respiratory rate, help reduce airway pressures in attempts to limit flow through the fistula, thus allowing it to heal. However, in patients who have ARDS, conservative measures are extremely difficult [12]. The main goal is to prevent hypoxia with acceptable ventilation. High frequency ventilation is occasionally applied, but this has limited utility if the lung parenchyma is not normal or BPF is distally located [13]. When conservative measures fail, the next approach is surgical intervention; this leads to closure of 80–95% of BPFs in patients without ARDS [14]. However, most patients who are on mechanical ventilation within the intensive care unit have multiple comorbidities and organ dysfunctions that usually preclude surgical options. The three reported patients were not surgical candidates. Their persistent air leaks affected oxygenation and carbon dioxide elimination and prevented extubation. Patients on mechanical ventilation with severe ARDS and high airway pressures have a very low likelihood of BPF resolution as long as they remain intubated [15]. Closure of the BPFs has helped our patients with mechanical extubation when conservative measures have failed. Although we waited for more than the five recommended days on each patient, an earlier intervention in certain patients with ARDS might be warranted to help shorten the duration of mechanical ventilation. Treating large BPFs early in the course of the disease might alter the outcome in patients with ARDS and reduce hospital length of stay. Preventing loss of PEEP and effective tidal volume can be lung protective by affecting lung recruitment, functional residual capacity, and prevention of atelectasis. This should protect lungs from ventilator-induced lung injury in addition to the benefit of improving oxygenation and carbon dioxide clearance. It should also be noted that our case series indicates safety in implanting the endobronchial valves in patients with ARDS. One other potential benefit is the potential reduction in cost of care. Although a formal financial analysis cannot be performed due to the small number of patients, we believe early intervention can be cost saving too due to reduction in ICU and ventilator days. Larger studies in this group of patients are required to further analyze safety and cost.

## 6. Conclusion

Data and literature regarding treatment for BPF in patients in the critical care unit with acute respiratory distress syndrome are limited. These cases will add to the literature regarding the application of endobronchial valves for patients with acute respiratory distress syndrome with persistent large volume air leaks who are unable to be weaned off the mechanical ventilator. In our opinion, patients with ARDS and large BPFs should be evaluated early in the course of the disease for intervention to close their fistulas with endobronchial valve placement. This may need to be formally evaluated in a prospective manner.
